# Supplementary material for: The role of state feelings of loneliness in the situational regulation of social affiliative behavior: Exploring the regulatory relations within a multilevel framework
Source: PLoS One. 2021 Jun 15;16(6):e0252775. doi: 10.1371/journal.pone.0252775 (PMC8205143; doi:10.1371/journal.pone.0252775)
Supplement: S1 File — (PDF) [file pone.0252775.s001.pdf]

Model Step 1 - ML

The Mixed Procedure

Model Information	
Data Set	CONSTUDY.PLOS_ONE
Dependent Variable	CON
Covariance Structures	Unstructured, Spatial Power
Subject Effects	ID, ID
Estimation Method	ML
Residual Variance Method	Profile
Fixed Effects SE Method	Kenward-Roger
Degrees of Freedom Method	Kenward-Roger

Dimensions	
Covariance Parameters	3
Columns in X	1
Columns in Z per Subject	1
Subjects	65
Max Obs per Subject	61

Number of Observations	
Number of Observations Read	3341
Number of Observations Used	3341
Number of Observations Not Used	0

Iteration History

Iteration	Evaluations	-2 Log Like	Criterion
0	1	33391.64164868	
1	3	32861.13562328	0.01207168
2	2	32702.95847185	0.00417967
3	1	32639.37639239	0.00030441
4	1	32635.10973807	0.00000295
5	1	32635.07015725	0.00000000

Convergence criteria met.

Covariance Parameter Estimates

Cov Parm	Subject	Estimate	Standard Error	Z Value	Pr Z	Alpha	Lower	Upper
UN(1,1)	ID	145.02	32.0988	4.52	<.0001	0.05	98.1016	236.08
SP(POW)	ID	0.6432	0.01416	45.43	<.0001	0.05	0.6154	0.6709
Residual		1136.71	31.9869	35.54	<.0001	0.05	1076.54	1202.11

Fit Statistics	
-2 Log Likelihood	32635.1
AIC (Smaller is Better)	32643.1
AICC (Smaller is Better)	32643.1
BIC (Smaller is Better)	32651.8

Null Model Likelihood Ratio Test

DF	Chi-Square	Pr > ChiSq
2	756.57	<.0001

Solution for Fixed Effects

Effect	Estimate	Standard Error	DF	t Value	Pr > |t|	Alpha	Lower	Upper

Solution for Fixed Effects								
Effect	Estimate	Standard Error	DF	t Value	Pr > |t|	Alpha	Lower	Upper
Intercept	57.8259	1.6773	65.1	34.48	<.0001	0.05	54.4762	61.1755

The Mixed Procedure

Model Step 2 - ML

Model Information	
Data Set	CONSTUDY.PLOS_ONE
Dependent Variable	CON
Covariance Structures	Unstructured, Spatial Power
Subject Effects	ID, ID
Estimation Method	ML
Residual Variance Method	Profile
Fixed Effects SE Method	Kenward-Roger
Degrees of Freedom Method	Kenward-Roger

Dimensions	
Covariance Parameters	3
Columns in X	12
Columns in Z per Subject	1
Subjects	65
Max Obs per Subject	61

Number of Observations	
Number of Observations Read	3341
Number of Observations Used	3341
Number of Observations Not Used	0

Iteration History			
Iteration	Evaluations	-2 Log Like	Criterion
0	1	32476.37318011	
1	2	32737.40406071	0.01176134
2	2	32549.26068218	0.00709221
3	1	32428.45268789	0.00320766
4	2	32376.61810972	0.00068718
5	2	32366.20485608	0.00006553
6	1	32365.25954176	0.00000207
7	1	32365.23161551	0.00000000

Convergence criteria met.

Covariance Parameter Estimates								
Cov Parm	Subject	Estimate	Standard Error	Z Value	Pr Z	Alpha	Lower	Upper
UN(1,1)	ID	69.5340	16.3513	4.25	<.0001	0.05	46.0232	117.16
SP(POW)	ID	0.2167	0.06441	3.36	0.0008	0.05	0.09042	0.3429
Residual		918.43	22.8257	40.24	<.0001	0.05	875.28	964.86

Fit Statistics	
-2 Log Likelihood	32365.2
AIC (Smaller is Better)	32395.2
AICC (Smaller is Better)	32395.4
BIC (Smaller is Better)	32427.8

Null Model Likelihood Ratio Test		
DF	Chi-Square	Pr > ChiSq
2	111.14	<.0001

Solution for Fixed Effects								
Effect	Estimate	Standard Error	DF	t Value	Pr > |t|	Alpha	Lower	Upper
Intercept	41.6747	1.9455	362	21.42	<.0001	0.05	37.8489	45.5006
tp	-0.01398	0.03594	914	-0.39	0.6975	0.05	-0.08451	0.05656
dh	-1.2546	1.1268	2873	-1.11	0.2656	0.05	-3.4639	0.9548
prev_CON	0.2653	0.01969	2849	13.47	<.0001	0.05	0.2267	0.3039
we	-9.0525	2.2708	1628	-3.99	<.0001	0.05	-13.5065	-4.5986
p_lone_cw	-0.1441	0.06649	3251	-2.17	0.0303	0.05	-0.2745	-0.01373
p_aff_cw	-0.1080	0.03606	2953	-2.99	0.0028	0.05	-0.1787	-0.03728
prev_CON*p_lone_cw	-0.00256	0.000913	3312	-2.80	0.0051	0.05	-0.00435	-0.00077
prev_CON*we	0.1910	0.03346	2119	5.71	<.0001	0.05	0.1254	0.2567
p_lone_cw*p_lone_cw	0.003077	0.001239	3241	2.48	0.0130	0.05	0.000648	0.005505
p_lone_cb	0.01863	0.1271	58.1	0.15	0.8840	0.05	-0.2357	0.2730
p_aff_cb	-0.1030	0.1154	57.8	-0.89	0.3759	0.05	-0.3341	0.1281

Type 3 Tests of Fixed Effects				
Effect	Num DF	Den DF	F Value	Pr > F
tp	1	914	0.15	0.6975
dh	1	2873	1.24	0.2656
prev_CON	1	2849	181.52	<.0001
we	1	1628	15.89	<.0001
p_lone_cw	1	3251	4.70	0.0303
p_aff_cw	1	2953	8.97	0.0028
prev_CON*p_lone_cw	1	3312	7.85	0.0051
prev_CON*we	1	2119	32.59	<.0001
p_lone_cw*p_lone_cw	1	3241	6.17	0.0130
p_lone_cb	1	58.1	0.02	0.8840
p_aff_cb	1	57.8	0.80	0.3759

The Mixed Procedure

Model Step 3 - ML

Model Information	
Data Set	CONSTUDY.PLOS_ONE
Dependent Variable	CON
Covariance Structures	Unstructured, Spatial Power
Subject Effects	ID, ID
Estimation Method	ML
Residual Variance Method	Profile
Fixed Effects SE Method	Kenward-Roger
Degrees of Freedom Method	Kenward-Roger

Dimensions	
Covariance Parameters	3
Columns in X	16
Columns in Z per Subject	1
Subjects	65
Max Obs per Subject	61

Number of Observations

Number of Observations	
Number of Observations Read	3341
Number of Observations Used	3341
Number of Observations Not Used	0

Iteration History			
Iteration	Evaluations	-2 Log Like	Criterion
0	1	32426.34887950	
1	2	32727.24593260	0.01199442
2	3	32556.40947014	.
3	1	32390.27664526	0.00235525
4	1	32354.20784352	0.00027982
5	1	32350.06181208	0.00001673
6	1	32349.83083717	0.00000013
7	1	32349.82915123	0.00000000

Convergence criteria met.

Covariance Parameter Estimates								
Cov Parm	Subject	Estimate	Standard Error	Z Value	Pr Z	Alpha	Lower	Upper
UN(1,1)	ID	50.7619	12.8721	3.94	<.0001	0.05	32.6474	89.6255
SP(POW)	ID	0.2171	0.06443	3.37	0.0008	0.05	0.09083	0.3434
Residual		918.45	22.8279	40.23	<.0001	0.05	875.30	964.89

Fit Statistics	
-2 Log Likelihood	32349.8
AIC (Smaller is Better)	32387.8
AICC (Smaller is Better)	32388.1
BIC (Smaller is Better)	32429.1

Null Model Likelihood Ratio Test		
DF	Chi-Square	Pr > ChiSq
2	76.52	<.0001

Solution for Fixed Effects								
Effect	Estimate	Standard Error	DF	t Value	Pr > |t|	Alpha	Lower	Upper
Intercept	42.5986	2.0008	326	21.29	<.0001	0.05	38.6625	46.5347
tp	-0.01671	0.03593	911	-0.47	0.6419	0.05	-0.08722	0.05379
dh	-1.2048	1.1260	2868	-1.07	0.2847	0.05	-3.4127	1.0031
prev_CON	0.2641	0.01970	2853	13.41	<.0001	0.05	0.2255	0.3028
we	-9.0266	2.2688	1623	-3.98	<.0001	0.05	-13.4768	-4.5764
p_lone_cw	-0.1424	0.06642	3253	-2.14	0.0321	0.05	-0.2727	-0.01222
p_aff_cw	-0.1088	0.03606	2952	-3.02	0.0026	0.05	-0.1795	-0.03806
prev_CON*p_lone_cw	-0.00255	0.000912	3321	-2.80	0.0052	0.05	-0.00434	-0.00076
prev_CON*we	0.1906	0.03342	2114	5.70	<.0001	0.05	0.1251	0.2562
p_lone_cw*p_lone_cw	0.002960	0.001236	3219	2.40	0.0167	0.05	0.000537	0.005382
p_lone_cb	0.1620	0.1209	57.9	1.34	0.1855	0.05	-0.08002	0.4041
p_aff_cb	-0.3389	0.1251	57.9	-2.71	0.0089	0.05	-0.5893	-0.08846
Gender	-3.6274	2.9711	59.1	-1.22	0.2270	0.05	-9.5723	2.3175
c_Age	-0.3492	0.3482	57.5	-1.00	0.3201	0.05	-1.0463	0.3478
c_MEF	-0.2092	0.06447	59.6	-3.24	0.0019	0.05	-0.3381	-0.08020
c_phq_9	0.6841	0.3470	57.8	1.97	0.0535	0.05	-0.01066	1.3788

Type 3 Tests of Fixed Effects				
Effect	Num DF	Den DF	F Value	Pr > F
tp	1	911	0.22	0.6419
dh	1	2868	1.14	0.2847
prev_CON	1	2853	179.82	<.0001
we	1	1623	15.83	<.0001
p_lone_cw	1	3253	4.60	0.0321
p_aff_cw	1	2952	9.10	0.0026
prev_CON*p_lone_cw	1	3321	7.83	0.0052
prev_CON*we	1	2114	32.52	<.0001
p_lone_cw*p_lone_cw	1	3219	5.74	0.0167
p_lone_cb	1	57.9	1.80	0.1855
p_aff_cb	1	57.9	7.34	0.0089
Gender	1	59.1	1.49	0.2270
c_Age	1	57.5	1.01	0.3201
c_MEF	1	59.6	10.53	0.0019
c_phq_9	1	57.8	3.89	0.0535

The Mixed Procedure

Model Step 3 - REML

Model Information	
Data Set	CONSTUDY.PLOS_ONE
Dependent Variable	CON
Covariance Structures	Unstructured, Spatial Power
Subject Effects	ID, ID
Estimation Method	REML
Residual Variance Method	Profile
Fixed Effects SE Method	Kenward-Roger
Degrees of Freedom Method	Kenward-Roger

Dimensions	
Covariance Parameters	3
Columns in X	16
Columns in Z per Subject	1
Subjects	65
Max Obs per Subject	61

Number of Observations	
Number of Observations Read	3341
Number of Observations Used	3341
Number of Observations Not Used	0

Iteration History			
Iteration	Evaluations	-2 Res Log Like	Criterion
0	1	32485.48142751	
1	2	32764.99218939	0.01171470
2	3	32598.02280683	.
3	1	32436.06164236	0.00208366
4	1	32403.90671714	0.00028855
5	1	32399.52499278	0.00002886
6	1	32399.10861602	0.00000090
7	1	32399.09652326	0.00000000

Convergence criteria met.

Covariance Parameter Estimates								
Cov Parm	Subject	Estimate	Standard Error	Z Value	Pr Z	Alpha	Lower	Upper
UN(1,1)	ID	60.3534	15.4674	3.90	<.0001	0.05	38.6546	107.30
SP(POW)	ID	0.2310	0.06247	3.70	0.0002	0.05	0.1085	0.3534
Residual		921.40	22.9753	40.10	<.0001	0.05	877.98	968.15

Fit Statistics	
-2 Res Log Likelihood	32399.1
AIC (Smaller is Better)	32405.1
AICC (Smaller is Better)	32405.1
BIC (Smaller is Better)	32411.6

Null Model Likelihood Ratio Test		
DF	Chi-Square	Pr > ChiSq
2	86.38	<.0001

Solution for Fixed Effects								
Effect	Estimate	Standard Error	DF	t Value	Pr > |t|	Alpha	Lower	Upper
Intercept	42.9264	2.0596	264	20.84	<.0001	0.05	38.8711	46.9817
tp	-0.01621	0.03615	866	-0.45	0.6540	0.05	-0.08717	0.05475
dh	-1.1986	1.1296	2851	-1.06	0.2887	0.05	-3.4135	1.0163
prev_CON	0.2578	0.01977	2836	13.03	<.0001	0.05	0.2190	0.2965
we	-8.9837	2.2800	1572	-3.94	<.0001	0.05	-13.4558	-4.5116
p_lone_cw	-0.1443	0.06658	3238	-2.17	0.0302	0.05	-0.2749	-0.01379
p_aff_cw	-0.1097	0.03614	2933	-3.03	0.0024	0.05	-0.1805	-0.03879
prev_CON*p_lone_cw	-0.00256	0.000913	3305	-2.80	0.0051	0.05	-0.00435	-0.00077
prev_CON*we	0.1906	0.03357	2071	5.68	<.0001	0.05	0.1248	0.2564
p_lone_cw*p_lone_cw	0.002986	0.001240	3213	2.41	0.0161	0.05	0.000554	0.005417
p_lone_cb	0.1621	0.1291	52.3	1.25	0.2151	0.05	-0.09703	0.4211
p_aff_cb	-0.3411	0.1336	52.3	-2.55	0.0136	0.05	-0.6092	-0.07306
Gender	-3.6638	3.1708	53.3	-1.16	0.2530	0.05	-10.0228	2.6952
c_Age	-0.3478	0.3719	52	-0.94	0.3540	0.05	-1.0941	0.3984
c_MEF	-0.2108	0.06878	53.6	-3.06	0.0034	0.05	-0.3487	-0.07288
c_phq_9	0.6911	0.3706	52.2	1.86	0.0679	0.05	-0.05258	1.4348

Type 3 Tests of Fixed Effects				
Effect	Num DF	Den DF	F Value	Pr > F
tp	1	866	0.20	0.6540
dh	1	2851	1.13	0.2887
prev_CON	1	2836	169.91	<.0001
we	1	1572	15.53	<.0001
p_lone_cw	1	3238	4.70	0.0302
p_aff_cw	1	2933	9.21	0.0024
prev_CON*p_lone_cw	1	3305	7.85	0.0051
prev_CON*we	1	2071	32.23	<.0001
p_lone_cw*p_lone_cw	1	3213	5.80	0.0161
p_lone_cb	1	52.3	1.57	0.2151
p_aff_cb	1	52.3	6.52	0.0136
Gender	1	53.3	1.34	0.2530
c_Age	1	52	0.87	0.3540
c_MEF	1	53.6	9.39	0.0034
c_phq_9	1	52.2	3.48	0.0679

The Mixed Procedure

Model Step 4 - ML

Model Information	
Data Set	CONSTUDY.PLOS_ONE
Dependent Variable	CON
Covariance Structures	Unstructured, Spatial Power
Subject Effects	ID, ID
Estimation Method	ML
Residual Variance Method	Profile
Fixed Effects SE Method	Kenward-Roger
Degrees of Freedom Method	Kenward-Roger

Dimensions	
Covariance Parameters	12
Columns in X	16
Columns in Z per Subject	4
Subjects	65
Max Obs per Subject	61

Number of Observations	
Number of Observations Read	3341
Number of Observations Used	3341
Number of Observations Not Used	0

Iteration History

Iteration	Evaluations	-2 Log Like	Criterion
0	1	32426.34887950	
1	3	33043.44074779	10050.758603
2	1	33036.86774479	6167.7738975
3	1	32984.78310326	1491.1438261
4	1	32776.91059755	0.01493957
5	3	32578.05067063	.
6	1	32427.75781793	.
7	3	32340.53918414	.
8	1	32326.88021105	.
9	1	32316.89980826	0.00016475
10	1	32314.32208527	0.00002557
11	1	32313.95023617	0.00000096
12	1	32313.93734134	0.00000000

Convergence criteria met.

Covariance Parameter Estimates

Cov Parm	Subject	Estimate	Standard Error	Z Value	Pr Z	Alpha	Lower	Upper
UN(1,1)	ID	76.9250	24.3319	3.16	0.0008	0.05	45.0202	160.45
UN(2,1)	ID	-21.5149	17.4161	-1.24	0.2167	0.05	-55.6499	12.6200
UN(2,2)	ID	32.9677	19.8778	1.66	0.0486	0.05	13.2862	176.37
UN(3,1)	ID	-21.6863	20.4998	-1.06	0.2901	0.05	-61.8653	18.4926
UN(3,2)	ID	-4.1931	16.6605	-0.25	0.8013	0.05	-36.8471	28.4609
UN(3,3)	ID	73.5493	32.4017	2.27	0.0116	0.05	36.2234	221.73
UN(4,1)	ID	0.3184	0.5383	0.59	0.5542	0.05	-0.7366	1.3735
UN(4,2)	ID	-0.03406	0.4934	-0.07	0.9450	0.05	-1.0012	0.9330

Covariance Parameter Estimates								
Cov Parm	Subject	Estimate	Standard Error	Z Value	Pr Z	Alpha	Lower	Upper
UN(4,3)	ID	0.7033	0.6100	1.15	0.2489	0.05	-0.4922	1.8988
UN(4,4)	ID	0.05799	0.02258	2.57	0.0051	0.05	0.03059	0.1492
SP(POW)	ID	0.2300	0.06472	3.55	0.0004	0.05	0.1032	0.3569
Residual		881.88	22.6647	38.91	<.0001	0.05	839.09	928.04

Fit Statistics	
-2 Log Likelihood	32313.9
AIC (Smaller is Better)	32369.9
AICC (Smaller is Better)	32370.4
BIC (Smaller is Better)	32430.8

Null Model Likelihood Ratio Test		
DF	Chi-Square	Pr > ChiSq
11	112.41	<.0001

Solution for Fixed Effects								
Effect	Estimate	Standard Error	DF	t Value	Pr > |t|	Alpha	Lower	Upper
Intercept	43.4865	2.1344	198	20.37	<.0001	0.05	39.2775	47.6956
tp	-0.02175	0.03659	832	-0.59	0.5525	0.05	-0.09358	0.05008
dh	-1.2082	1.3291	63.9	-0.91	0.3667	0.05	-3.8634	1.4470
prev_CON	0.2502	0.01993	2788	12.55	<.0001	0.05	0.2111	0.2893
we	-6.3796	2.6195	201	-2.44	0.0157	0.05	-11.5449	-1.2143
p_lone_cw	-0.1368	0.07704	283	-1.78	0.0769	0.05	-0.2884	0.01487
p_aff_cw	-0.1186	0.03638	2952	-3.26	0.0011	0.05	-0.1900	-0.04732
prev_CON*p_lone_cw	-0.00282	0.000965	2238	-2.92	0.0035	0.05	-0.00471	-0.00093
prev_CON*we	0.1528	0.03553	1066	4.30	<.0001	0.05	0.08312	0.2225
p_lone_cw*p_lone_cw	0.002932	0.001368	1199	2.14	0.0323	0.05	0.000248	0.005615
p_lone_cb	0.1232	0.1308	56.5	0.94	0.3504	0.05	-0.1388	0.3851
p_aff_cb	-0.3370	0.1347	55.4	-2.50	0.0153	0.05	-0.6068	-0.06709
Gender	-4.1804	3.1849	56.6	-1.31	0.1946	0.05	-10.5590	2.1981
c_Age	-0.2192	0.3738	56.5	-0.59	0.5598	0.05	-0.9678	0.5293
c_MEF	-0.2255	0.06925	57	-3.26	0.0019	0.05	-0.3642	-0.08684
c_phq_9	0.7282	0.3735	55.2	1.95	0.0563	0.05	-0.02018	1.4767

Type 3 Tests of Fixed Effects				
Effect	Num DF	Den DF	F Value	Pr > F
tp	1	832	0.35	0.5525
dh	1	63.9	0.83	0.3667
prev_CON	1	2788	157.48	<.0001
we	1	201	5.93	0.0157
p_lone_cw	1	283	3.15	0.0769
p_aff_cw	1	2952	10.64	0.0011
prev_CON*p_lone_cw	1	2238	8.54	0.0035
prev_CON*we	1	1066	18.50	<.0001
p_lone_cw*p_lone_cw	1	1199	4.59	0.0323
p_lone_cb	1	56.5	0.89	0.3504
p_aff_cb	1	55.4	6.26	0.0153
Gender	1	56.6	1.72	0.1946
c_Age	1	56.5	0.34	0.5598
c_MEF	1	57	10.60	0.0019
c_phq_9	1	55.2	3.80	0.0563

The Mixed Procedure

Model Step 4 - REML

Model Information	
Data Set	CONSTUDY.PLOS_ONE
Dependent Variable	CON
Covariance Structures	Unstructured, Spatial Power
Subject Effects	ID, ID
Estimation Method	REML
Residual Variance Method	Profile
Fixed Effects SE Method	Kenward-Roger
Degrees of Freedom Method	Kenward-Roger

Dimensions	
Covariance Parameters	12
Columns in X	16
Columns in Z per Subject	4
Subjects	65
Max Obs per Subject	61

Number of Observations	
Number of Observations Read	3341
Number of Observations Used	3341
Number of Observations Not Used	0

Iteration History

Iteration	Evaluations	-2 Res Log Like	Criterion
0	1	32485.48142751	
1	3	33079.83850140	53118.952869
2	1	33073.64206614	6266.8244846
3	1	33019.99864493	1526.9400078
4	1	32810.15820776	0.01471333
5	3	32626.51483240	.
6	1	32473.13765540	.
7	3	32391.08067120	.
8	1	32375.98443294	.
9	1	32364.68581062	0.00020329
10	1	32361.47155447	0.00003572
11	1	32360.94380920	0.00000178
12	1	32360.91957170	0.00000001

Convergence criteria met.

Covariance Parameter Estimates

Cov Parm	Subject	Estimate	Standard Error	Z Value	Pr Z	Alpha	Lower	Upper
UN(1,1)	ID	86.7145	26.8376	3.23	0.0006	0.05	51.2594	177.52
UN(2,1)	ID	-21.5890	18.1971	-1.19	0.2355	0.05	-57.2545	14.0766
UN(2,2)	ID	33.9044	20.2810	1.67	0.0473	0.05	13.7394	178.05
UN(3,1)	ID	-21.9663	21.9288	-1.00	0.3165	0.05	-64.9459	21.0133
UN(3,2)	ID	-4.6339	17.2846	-0.27	0.7886	0.05	-38.5110	29.2432
UN(3,3)	ID	80.1433	34.0478	2.35	0.0093	0.05	40.3019	229.91
UN(4,1)	ID	0.3206	0.5755	0.56	0.5775	0.05	-0.8073	1.4485
UN(4,2)	ID	-0.03897	0.5079	-0.08	0.9388	0.05	-1.0344	0.9565

Covariance Parameter Estimates								
Cov Parm	Subject	Estimate	Standard Error	Z Value	Pr Z	Alpha	Lower	Upper
UN(4,3)	ID	0.7250	0.6391	1.13	0.2566	0.05	-0.5277	1.9777
UN(4,4)	ID	0.06175	0.02362	2.61	0.0045	0.05	0.03289	0.1556
SP(POW)	ID	0.2452	0.06246	3.92	<.0001	0.05	0.1227	0.3676
Residual		883.59	22.7710	38.80	<.0001	0.05	840.61	929.98

Fit Statistics	
-2 Res Log Likelihood	32360.9
AIC (Smaller is Better)	32384.9
AICC (Smaller is Better)	32385.0
BIC (Smaller is Better)	32411.0

Null Model Likelihood Ratio Test		
DF	Chi-Square	Pr > ChiSq
11	124.56	<.0001

Solution for Fixed Effects								
Effect	Estimate	Standard Error	DF	t Value	Pr > |t|	Alpha	Lower	Upper
Intercept	43.8595	2.1938	175	19.99	<.0001	0.05	39.5296	48.1893
tp	-0.02141	0.03686	796	-0.58	0.5614	0.05	-0.09377	0.05094
dh	-1.2002	1.3370	62.9	-0.90	0.3728	0.05	-3.8721	1.4716
prev_CON	0.2431	0.02000	2796	12.15	<.0001	0.05	0.2039	0.2824
we	-6.0989	2.6536	198	-2.30	0.0226	0.05	-11.3320	-0.8659
p_lone_cw	-0.1400	0.07768	274	-1.80	0.0726	0.05	-0.2929	0.01294
p_aff_cw	-0.1202	0.03647	2952	-3.30	0.0010	0.05	-0.1917	-0.04869
prev_CON*p_lone_cw	-0.00281	0.000967	2260	-2.91	0.0037	0.05	-0.00471	-0.00092
prev_CON*we	0.1490	0.03577	1122	4.16	<.0001	0.05	0.07878	0.2191
p_lone_cw*p_lone_cw	0.002950	0.001374	1233	2.15	0.0320	0.05	0.000254	0.005646
p_lone_cb	0.1267	0.1401	51.3	0.90	0.3698	0.05	-0.1544	0.4079
p_aff_cb	-0.3394	0.1443	50.4	-2.35	0.0226	0.05	-0.6292	-0.04956
Gender	-4.2398	3.4101	51.5	-1.24	0.2194	0.05	-11.0844	2.6048
c_Age	-0.2278	0.4002	51.2	-0.57	0.5717	0.05	-1.0312	0.5755
c_MEF	-0.2263	0.07412	51.8	-3.05	0.0036	0.05	-0.3750	-0.07753
c_phq_9	0.7361	0.4002	50.3	1.84	0.0718	0.05	-0.06759	1.5399

Type 3 Tests of Fixed Effects				
Effect	Num DF	Den DF	F Value	Pr > F
tp	1	796	0.34	0.5614
dh	1	62.9	0.81	0.3728
prev_CON	1	2796	147.72	<.0001
we	1	198	5.28	0.0226
p_lone_cw	1	274	3.25	0.0726
p_aff_cw	1	2952	10.86	0.0010
prev_CON*p_lone_cw	1	2260	8.46	0.0037
prev_CON*we	1	1122	17.34	<.0001
p_lone_cw*p_lone_cw	1	1233	4.61	0.0320
p_lone_cb	1	51.3	0.82	0.3698
p_aff_cb	1	50.4	5.53	0.0226
Gender	1	51.5	1.55	0.2194
c_Age	1	51.2	0.32	0.5717
c_MEF	1	51.8	9.32	0.0036
c_phq_9	1	50.3	3.38	0.0718

The Mixed Procedure

Model Step 5 - ML

Model Information	
Data Set	CONSTUDY.PLOS_ONE
Dependent Variable	CON
Covariance Structures	Unstructured, Spatial Power
Subject Effects	ID, ID
Estimation Method	ML
Residual Variance Method	Profile
Fixed Effects SE Method	Kenward-Roger
Degrees of Freedom Method	Kenward-Roger

Dimensions	
Covariance Parameters	12
Columns in X	22
Columns in Z per Subject	4
Subjects	65
Max Obs per Subject	61

Number of Observations	
Number of Observations Read	3341
Number of Observations Used	3341
Number of Observations Not Used	0

Iteration History

Iteration	Evaluations	-2 Log Like	Criterion
0	1	32415.35797714	
1	2	33034.63866570	4985.6805084
2	1	33028.78305850	40986.308628
3	1	32991.84748536	6382.6341595
4	1	32808.65852650	0.01611899
5	3	32680.45091313	.
6	1	32480.07852334	.
7	1	32376.04440666	.
8	2	32324.30081879	.
9	1	32307.83547905	0.00015914
10	1	32305.46949121	0.00001089
11	1	32305.31824316	0.00000012
12	1	32305.31666531	0.00000000

Convergence criteria met.

Covariance Parameter Estimates

Cov Parm	Subject	Estimate	Standard Error	Z Value	Pr Z	Alpha	Lower	Upper
UN(1,1)	ID	74.3509	23.6009	3.15	0.0008	0.05	43.4425	155.56
UN(2,1)	ID	-19.6023	17.0338	-1.15	0.2498	0.05	-52.9880	13.7833
UN(2,2)	ID	31.0190	19.5485	1.59	0.0563	0.05	12.1169	184.90
UN(3,1)	ID	-20.1880	20.2285	-1.00	0.3183	0.05	-59.8351	19.4591
UN(3,2)	ID	-3.9185	16.4887	-0.24	0.8122	0.05	-36.2357	28.3987
UN(3,3)	ID	72.4351	32.1826	2.25	0.0122	0.05	35.4989	221.00
UN(4,1)	ID	-0.00511	0.5275	-0.01	0.9923	0.05	-1.0390	1.0288
UN(4,2)	ID	-0.05809	0.4768	-0.12	0.9030	0.05	-0.9926	0.8764

Covariance Parameter Estimates								
Cov Parm	Subject	Estimate	Standard Error	Z Value	Pr Z	Alpha	Lower	Upper
UN(4,3)	ID	1.3384	0.6687	2.00	0.0453	0.05	0.02787	2.6490
UN(4,4)	ID	0.05077	0.02163	2.35	0.0094	0.05	0.02549	0.1462
SP(POW)	ID	0.2323	0.06446	3.60	0.0003	0.05	0.1059	0.3586
Residual		882.16	22.6987	38.86	<.0001	0.05	839.31	928.40

Fit Statistics	
-2 Log Likelihood	32305.3
AIC (Smaller is Better)	32373.3
AICC (Smaller is Better)	32374.0
BIC (Smaller is Better)	32447.2

Null Model Likelihood Ratio Test		
DF	Chi-Square	Pr > ChiSq
11	110.04	<.0001

Information Criteria						
Neg2LogLike	Parms	AIC	AICC	HQIC	BIC	CAIC
32305.3	34	32373.3	32374.0	32402.5	32447.2	32481.2

Solution for Fixed Effects								
Effect	Estimate	Standard Error	DF	t Value	Pr > |t|	Alpha	Lower	Upper
Intercept	43.3075	2.1256	201	20.37	<.0001	0.05	39.1162	47.4988
tp	-0.02082	0.03653	818	-0.57	0.5689	0.05	-0.09253	0.05089
dh	-1.1781	1.3172	63.7	-0.89	0.3745	0.05	-3.8097	1.4535
prev_CON	0.2485	0.01995	2810	12.46	<.0001	0.05	0.2094	0.2876
we	-6.6223	2.6145	201	-2.53	0.0121	0.05	-11.7777	-1.4669
p_lone_cw	-0.1981	0.08502	279	-2.33	0.0205	0.05	-0.3655	-0.03074
p_aff_cw	-0.1205	0.03639	2938	-3.31	0.0009	0.05	-0.1919	-0.04916
prev_CON*p_lone_cw	-0.00270	0.000969	2309	-2.79	0.0053	0.05	-0.00460	-0.00080
prev_CON*we	0.1559	0.03543	1048	4.40	<.0001	0.05	0.08636	0.2254
p_lone_cw*p_lone_cw	0.003596	0.001419	2328	2.53	0.0114	0.05	0.000813	0.006380
p_lone_cb	0.1581	0.1294	56.6	1.22	0.2270	0.05	-0.1011	0.4173
p_aff_cb	-0.3569	0.1337	56.3	-2.67	0.0099	0.05	-0.6247	-0.08921
Gender	-3.9048	3.1724	57.3	-1.23	0.2234	0.05	-10.2568	2.4472
c_Age	-0.3050	0.3728	56.4	-0.82	0.4168	0.05	-1.0518	0.4418
c_MEF	-0.2134	0.06880	57.7	-3.10	0.0030	0.05	-0.3512	-0.07569
c_phq_9	0.7148	0.3712	56.3	1.93	0.0592	0.05	-0.02866	1.4583
p_lone_cw*c_MEF	0.002994	0.002674	46.7	1.12	0.2686	0.05	-0.00239	0.008375
p_lone_cw*c_phq_9	0.000159	0.01425	47.3	0.01	0.9912	0.05	-0.02850	0.02882
p_lone_cw*Gender	0.1024	0.1346	53.6	0.76	0.4500	0.05	-0.1675	0.3724
p_lone_cw*c_Age	-0.02652	0.01906	75.5	-1.39	0.1682	0.05	-0.06448	0.01145
p_lone_cw*p_aff_cb	-0.00268	0.005054	42.3	-0.53	0.5984	0.05	-0.01288	0.007516
p_lone_cw*p_lone_cb	0.008999	0.004841	43.2	1.86	0.0699	0.05	-0.00076	0.01876

Type 3 Tests of Fixed Effects				
Effect	Num DF	Den DF	F Value	Pr > F
tp	1	818	0.32	0.5689
dh	1	63.7	0.80	0.3745
prev_CON	1	2810	155.15	<.0001
we	1	201	6.42	0.0121
p_lone_cw	1	279	5.43	0.0205

Type 3 Tests of Fixed Effects				
**Effect**	**Num DF**	**Den DF**	**F Value**	**Pr > F**
p_aff_cw	1	2938	10.97	0.0009
prev_CON*p_lone_cw	1	2309	7.79	0.0053
prev_CON*we	1	1048	19.36	<.0001
p_lone_cw*p_lone_cw	1	2328	6.42	0.0114
p_lone_cb	1	56.6	1.49	0.2270
p_aff_cb	1	56.3	7.13	0.0099
Gender	1	57.3	1.51	0.2234
c_Age	1	56.4	0.67	0.4168
c_MEF	1	57.7	9.62	0.0030
c_phq_9	1	56.3	3.71	0.0592
p_lone_cw*c_MEF	1	46.7	1.25	0.2686
p_lone_cw*c_phq_9	1	47.3	0.00	0.9912
p_lone_cw*Gender	1	53.6	0.58	0.4500
p_lone_cw*c_Age	1	75.5	1.94	0.1682
p_lone_cw*p_aff_cb	1	42.3	0.28	0.5984
p_lone_cw*p_lone_cb	1	43.2	3.46	0.0699
