# Supplementary material for: The role of state feelings of loneliness in the situational regulation of social affiliative behavior: Exploring the regulatory relations within a multilevel framework
Source: PLoS One. 2021 Jun 15;16(6):e0252775. doi: 10.1371/journal.pone.0252775 (PMC8205143; doi:10.1371/journal.pone.0252775)
Supplement: S2 File — (PDF) [file pone.0252775.s002.pdf]

```
****Model Step 1****;
proc mixed data=fgastudy.red_fga noclprint PLOTS(MAXPOINTS=6000) method=ml
covtest cl;
class ID;
model CON= / ddfm=kr solution s CL;
random intercept / type=un sub=ID;
repeated / type=SP(POW)(conttime2) sub=ID;


****Model Step 2****;
proc mixed data =fgastudy.red_fga noclprint PLOTS(MAXPOINTS=6000) method=ml
covtest cl;
class ID;
model CON= tp dh prev_Con we p_lone_cw p_aff_cw prev_Con*p_lone_cw prev_CON*we
p_lone_cw*p_lone_cw p_lone_cb p_aff_cb / ddfm=kr solution s CL;
random intercept / type=un sub=ID;
repeated / type=SP(POW)(conttime2) sub=ID;


****Model Step 3****;
proc mixed data =fgastudy.red_fga noclprint PLOTS(MAXPOINTS=6000) method=ml
covtest cl;
class ID;
model CON= tp dh prev_Con we p_lone_cw p_aff_cw prev_Con*p_lone_cw prev_CON*we
p_lone_cw*p_lone_cw p_lone_cb p_aff_cb gender c_age c_MEF c_phq_9 / ddfm=kr
solution s CL;
random intercept / type=un sub=ID;
repeated / type=SP(POW)(conttime2) sub=ID;

*REML*;
proc mixed data =fgastudy.red_fga noclprint PLOTS(MAXPOINTS=6000) method=reml
covtest cl;
class ID;
model CON= tp dh prev_Con we p_lone_cw p_aff_cw prev_Con*p_lone_cw prev_CON*we
p_lone_cw*p_lone_cw p_lone_cb p_aff_cb gender c_age c_MEF c_phq_9 / ddfm=kr
solution s CL;
random intercept / type=un sub=ID;
repeated / type=SP(POW)(conttime2) sub=ID;


****Model Step 4****;
proc mixed data =fgastudy.red_fga noclprint PLOTS(MAXPOINTS=6000) method=ml
covtest cl;
class ID;
model CON= tp dh prev_Con we p_lone_cw p_aff_cw prev_Con*p_lone_cw prev_CON*we
p_lone_cw*p_lone_cw p_lone_cb p_aff_cb gender c_age c_MEF c_phq_9 / ddfm=kr
solution outpm=pred s CL;
random intercept dh we p_lone_cw / type=un sub=ID;
repeated / type=SP(POW)(conttime2) sub=ID;

*REML*;
proc mixed data =fgastudy.red_fga noclprint PLOTS(MAXPOINTS=6000) method=reml
covtest cl;
class ID;
```

```
model CON= tp dh prev_Con we p_lone_cw p_aff_cw prev_Con*p_lone_cw prev_CON*we
p_lone_cw*p_lone_cw p_lone_cb p_aff_cb gender c_age c_MEF c_phq_9 / ddfm=kr
solution outpm=pred s CL;
random intercept dh we p_lone_cw / type=un sub=ID;
repeated / type=SP(POW)(conttime2) sub=ID;


****Model Step 5****;
proc mixed data =fgastudy.red_fga noclprint PLOTS(MAXPOINTS=6000) method=ml
covtest ic cl;
class ID;
model CON= tp dh prev_Con we p_lone_cw p_aff_cw prev_Con*p_lone_cw prev_CON*we
p_lone_cw*p_lone_cw
p_lone_cb p_aff_cb gender c_age c_MEF c_phq_9 p_lone_cw*c_MEF p_lone_cw*c_phq_9
p_lone_cw*gender p_lone_cw*c_age p_lone_cw*p_aff_cb p_lone_cw*p_lone_cb/ ddfm=kr
solution outpm=pred s CL;
random intercept dh we p_lone_cw / type=un sub=ID;
repeated / type=SP(POW)(conttime2) sub=ID;

*for R^2*;
proc mixed data =fgastudy.red_fga noclprint PLOTS(MAXPOINTS=6000) method=ml
covtest ic cl;
class ID;
model CON= tp dh prev_Con we p_lone_cw p_aff_cw prev_Con*p_lone_cw prev_CON*we
p_lone_cw*p_lone_cw
p_lone_cb p_aff_cb gender c_age c_MEF c_phq_9 p_lone_cw*c_MEF p_lone_cw*c_phq_9
p_lone_cw*gender p_lone_cw*c_age p_lone_cw*p_aff_cb p_lone_cw*p_lone_cb/ ddfm=kr
solution outpm=pred s CL;
random intercept / type=un sub=ID;
repeated / type=SP(POW)(conttime2) sub=ID;
```
